# Decreased EDHF‐mediated relaxation is a major mechanism in endothelial dysfunction in resistance arteries in aged mice on prolonged high‐fat sucrose diet

**DOI:** 10.14814/phy2.13502

**Published:** 2017-12-07

**Authors:** Shannon M. Dunn, Kumuda C. Das

**Affiliations:** ^1^ Department of Pharmacology & Neuroscience Texas Tech University Health Sciences Center Lubbock Texas; ^2^ The Department of Translational & Vascular Biology University of Texas Health Sciences Center at Tyler Tyler Texas; ^3^ Present address: Shannon Dunn 685 W. Baltimore Street, MSTF Room, 357‐B Baltimore Maryland

**Keywords:** Aging, endothelial dysfunction, endothelium‐dependent hyperpolarizing factor, high‐fat sucrose diet, obesity, peroxinitrite, reactive oxygen species, vascular relaxation

## Abstract

High‐fat sucrose (HFS) diet in aged individuals causes severe weight gain (obesity) with much higher risk of cardiovascular diseases such as hypertension or atherosclerosis. Endothelial dysfunction is a major contributor for these vascular disorders. We hypothesize that prolonged ingestion of HFS diet by aged mice would accentuate endothelial dysfunction in the small resistance arteries. Male C57BL/6J mice at 12 weeks of age were divided into four groups and fed either normal chow (NC) or high‐fat sucrose diet (HFS). Young group received NC for 4 months, and high‐fat diet (HFD) for 3 months and 1 month HFS + 10% Sucrose (HFS diet). Aged mice received NC for 12 months. Aged HFS group received HFD for 4 months + 1 month HFD + 10% sucrose + 8 months HFD. Total body weight, plasma blood glucose levels, and glucose tolerance were determined in all groups. Isolated mesenteric arteries were assessed for arterial remodeling, myogenic tone, and vasomotor responses using pressure and wire myography. Both young and aged HFS mice showed impaired glucose tolerance (Y‐NC, 137 ± 8.5 vs. Y‐NC HFS, 228 ± 11.71; A‐NC, 148 ± 6.42 vs. A‐HFS, 225 ± 10.99), as well as hypercholesterolemia (Y‐NC 99.50 ± 6.35 vs. Y‐HFS 220.40 ± 16.34 mg/dL; A‐NC 108.6 ± vs. A‐HFS 279 ± 21.64) and significant weight gain (Y‐NC 32.13 ± 0.8 g vs. Y‐HFS 47.87 ± 2.18 g; A‐NC 33.72 vs. A‐HFS 56.28 ± 3.47 g) compared to both groups of mice on NC. The mesenteric artery from mice with prolonged HFS diet resulted in outward hypertrophic remodeling, increased stiffness, reduced myogenic tone, impaired vasodilation, increased contractility and blunted nitric oxide (NO) and EDH‐mediated relaxations. Ebselen, a peroxinitrite scavenger rescued the endothelium derived relaxing factor (EDHF)‐mediated relaxations. Our findings suggest that prolonged diet‐induced obesity of aged mice can worsen small resistance artery endothelial dysfunction due to decrease in NO and EDHF‐mediated relaxation, but, EDHF‐mediated relaxation is a major contributor to overall endothelial dysfunction.

## Introduction

Obesity is linked to the onset of several cardiovascular diseases and metabolic complications such as hypertension, atherosclerosis, and Type‐2 diabetes (Li et al. 2017). In addition, obesity is also linked to the aging process, as a major proportion of aged individuals are obese (Bailey‐Downs et al. 2013). Consequently, the risk of cardiovascular and metabolic disorders is considerably higher in these individuals. Obesity‐mediated dysfunction of the vascular endothelium has been implicated as an underlying mechanism for all of these cardiovascular and metabolic disorders (Bailey‐Downs et al. 2013). Endothelial cells line the lumen of vascular tissue and secrete several active compounds that is essential to promote vascular flow and maintain healthy arterial structure and function. However, normal function of endothelium is impaired in obese individuals (Sloboda et al. 2012). In addition, hyperglycemia induces endothelial dysfunction, which is a major vascular complication in diabetes (Sharma et al. 2017). This dysfunction of endothelium also occurs in aged individuals (Seals et al. 2011). Endothelium‐dependent vasodilation has been shown to be impaired after 40 years of age and progressively decline with increasing age (Seals et al. 2011).

Endothelial dysfunction is primarily characterized by an impaired endothelium‐dependent dilation due to a reduction in NO bioavailability (Hamilton et al. 2002; Crabtree et al. 2013). In obesity NO production decreases due to increased oxidative stress and inflammation (Bailey‐Downs et al. 2013). In hyperglycemia EC apoptosis occurs, which has been shown to cause decreased NO production (Lan et al. 2015). Furthermore, during aging the eNOS becomes dysfunctional and produces superoxide anion (O_2_^•−^) instead of NO causing decreased NO availability (Hilgers et al. 2017). Endothelial dysfunction in small resistance arteries is an early marker of developing vascular disorders such as atherosclerosis (Hilgers and Das 2015). Small vessels contribute substantially to the vascular resistance, and are key determinant of impaired microcirculatory function (Bender et al. 2015). Impaired vasodilation has been shown to occur in peripheral vascular disease, diabetes, hypercholestromia, hypertension, chronic renal failure and atherosclerosis, aortic aneurism and other vascular complications (Abularrage et al. 2005). Endothelial dysfunction can also occur in a similar fashion in multiple vascular beds throughout the body (Higashi et al. 2012). However, the structural and functional differences in vascular beds critically impact aged‐related flow‐mediated dilation resulting in on set of cardiovascular diseases (CVD). For example, resistance artery relaxation is largely mediated by endothelium derived hyperpolarizing factor (EDHF), however, NO is the principal relaxing factor in large conduit arteries such as aortae (Matoba et al. 2000).

Impaired flow‐mediated dilation in young adults has been observed in obesity due to a “western‐type” high‐fat/high‐sucrose diet (Keogh et al. 2005). Studies using isolated arteries from animals on a HFD have shown endothelial dysfunction in both large conduit (Woodman et al. 2005) and small resistance arteries (Erdei et al. 2006; Donato et al. 2012). However, structural and functional alterations of resistance arteries in response to prolonged HFS in aging remains understudied. Both, obesity and aging are accompanied by arterial stiffening of large conduit arteries involving vascular remodeling processes including vascular cell proliferation, migration, hypertrophy, and changes in the composition of elastin/collagen in the extracellular matrix (Chatterjee et al. 2014). However, microvascular remodeling associated with obesity and insulin resistance is characterized by medial hypertrophy and increased extracellular matrix deposition, which may be vascular‐bed specific (Briones et al. 2014). Previous studies have shown that a high‐fat, low‐nutrient diet increases the likelihood and advancement of obesity and diabetes (Willett et al. 1990; Giovannucci and Willett 1994; Petrofsky et al. 2013). Obesity and aging are risk factors for developing Type II diabetes (T2D), with obesity being one of the strongest risk markers (Cassano et al. 1992; Chan et al. 1994; Carey et al. 1997). There are virtually no studies addressing the effects of obesity in aged resistance artery endothelial dysfunction. Furthermore, the specific mechanism of endothelial dysfunction in these arteries has not been addressed.

Endothelium derived relaxing factor has been shown to be important in modulating vasomotor tone in microvessels (Shimokawa et al. 1996; Urakami‐Harasawa et al. 1997). Although EDHF is very distinct from NO, studies suggest that both factors are functionally similar in maintaining vascular relaxations (Nagao et al. 1992). For example, both NO and EDHF are synthesized by the endothelial cells (Takaki et al. 2008). In conditions where NO‐mediated relaxation is decreased (e.g., hypertension, hyperlipidemia) EDHF compensates for NO to cause endothelium‐dependent relaxations (Takaki et al. 2008). For example, a dysfunctional nitric oxide synthase (eNOS) in aged animals produces O_2_^•−^ instead of NO due either to oxidation of Tetrahydrobiopterin (BH_4_) or depletion of L‐arginine (Chen et al. 2013). The O_2_^•−^ produced by eNOS is readily converted to H_2_O_2_, and H_2_O_2_ function as an EDHF in this setting (Matoba et al. 2000). Although endothelial dysfunction has been reported in aged animals and also due to HFD fed aged animals, the exact mechanism of endothelial dysfunction in these animals remains understudied. For example, although decreased NO production has been attributed to endothelial dysfunction due to HFD in aged mice, the role of EDHF in HFD fed mice and HFD fed aged mice remains unclear. In addition, the role of HFS diet or increased blood glucose in EDHF –mediated endothelial dysfunction remains poorly understood.

In this study we hypothesized that HFS diet in aged mice would promote endothelial dysfunction in the resistance arteries due to impairment of EDHF‐dependent vascular relaxation. Our study design incorporates both, an aging model and a diet‐induced obesity model. We utilized a mice model with near lifelong HFS diet consumption to evaluate age‐related vascular dysfunction. We studied the effects of HFD on bodyweight, glucose metabolism, cholesterol levels, epididymal white adipose tissue (EWAT) deposition, microvascular reactivity and structure in young and aged mice. We also evaluated whether HFS diet in aged mice resistance arteries would show endothelial dysfunction due to impairment of EDHF. We show here that HFS diet caused severe obesity, and impaired glucose tolerance in mice that resembles a T2D phenotype. Furthermore, HFS exacerbated age‐related endothelial dysfunction in the small resistance arteries that is dependent on EDHF‐mediated relaxation since loss of EDHF in the resistance arteries caused endothelial dysfunction due to HFS in aged mice.

## Materials and Methods

### Animals and treatments

**Figure 1:**
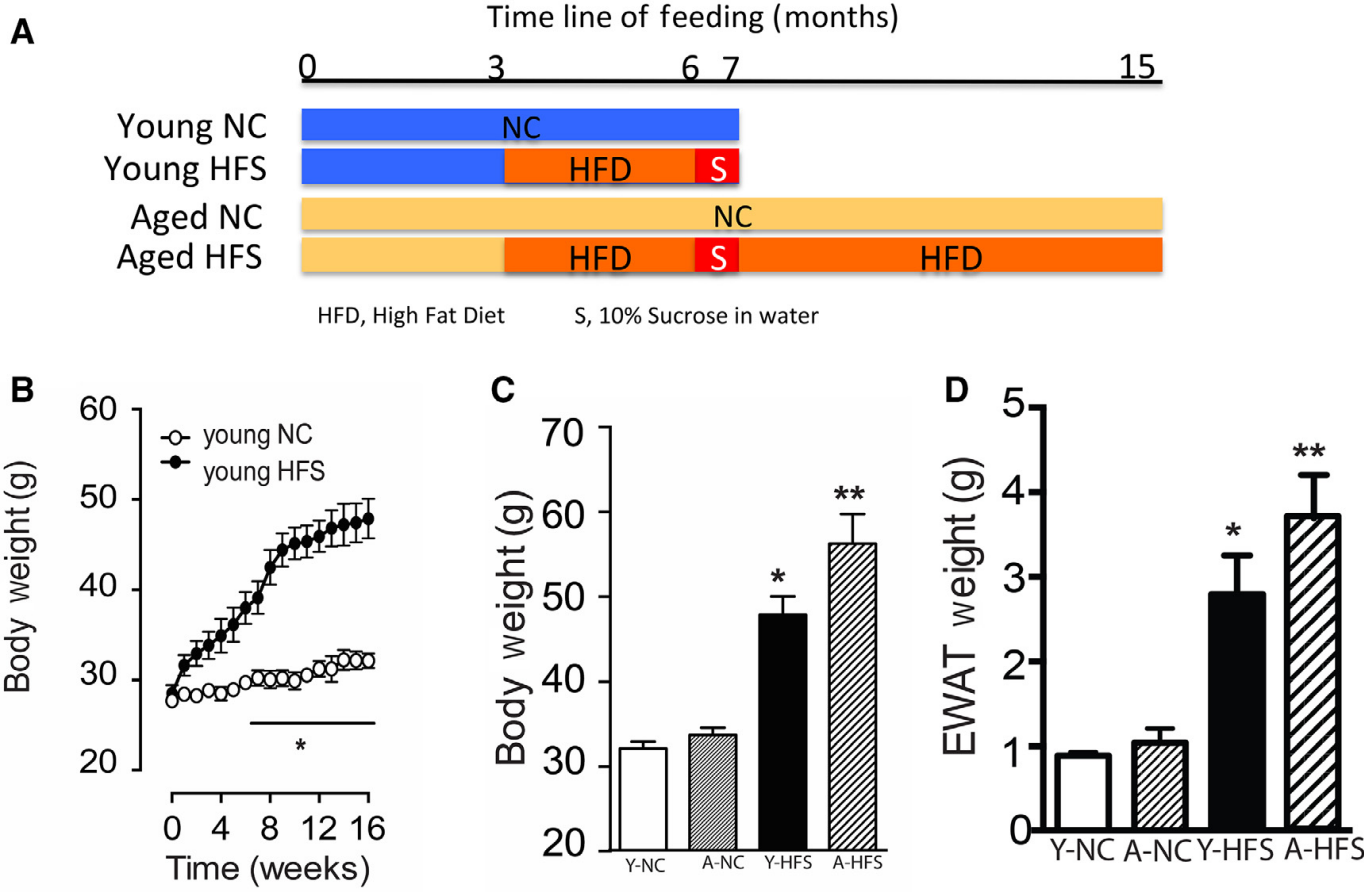
Effect of HFS on body weight of young and aged mice: Study timeline of feeding (A), weekly time log of body weight increases in young mice (B), final body weight of young and aged mice on NC or HFS (C), weight of epididymal fat pads for young and aged mice on NC or HFS (D), derived from mice receiving 6 months of normal chow (NC young; white bars), 16 months of NC (NC aged; dark gray bars), 3 months of a high‐fat diet followed by 4 weeks of 10% sucrose in drinking water (HFS young; black bars), and 12 months of HFS (HFS aged; black bar). Values are shown as means ± SEM (n = 6); *P <* *0.05 versus young NC; **P <* *0.05 versus aged NC.

Male C57BL/6J mice at 3 months were fed a normal chow (NC) diet (12% kcal from fat, Harlan Laboratories, Inc.; Cat# 2020X) or HFD (42% kcal from fat, Harlan Laboratories, Inc., Envigo, Cat #88137). Sucrose (10% diluted in tap water, Sigma Aldrich) was given in addition to the HFD for a period of 1 month in the HFS group. All mice enter into the study at 3 months age. Mice were divided into four groups; group 1, young mice with normal chow (NC) diet for 4 months; group 2, young mice with high‐fat sucrose (HFS) diet (3 months with HFD plus 1 month in 10% sucrose), group 3, aged (12 months) mice with NC, and group 4, aged mice with HFS diet (12 months). Mice in the young group were fed NC for 4 months or HFD for 3 months followed by 1 month of HFD plus 10% sucrose. Mice in the NC aging group were placed on a NC chow until an age of 15 months. Mice in the aged HFS group were placed on HFD for 3 months and HFD+ 10% sucrose for 1 month. These mice were further placed on HFD for an additional 8 months (Fig. 1A). Bodyweight was monitored weekly in the two young mice groups. All procedures were approved by the IACUC at the Texas Tech University Health Sciences Center and were consistent with the *Guide for the Care and Use of Laboratory Animals* published by the National Institute of Health. All animals were maintained on a standard 12‐h light/12‐h dark cycle, in a temperature‐controlled barrier facility.

### Fasting glucose and insulin measurement

Glucose and serum insulin levels were measured after 4–6 h of fasting from mice receiving 4 months of normal chow (NC young) or 12 months of NC (NC aged); 4 months of HFS (HFS young) or 12 months of HFS (HFS aged). The tail was snipped and fasting blood glucose levels were measured using the OneTouch Ultra glucose monitor (LifeScan Inc.). Blood was drawn from the orbital sinus, spun down at 3000***g*** for 15 min and serum was separated and insulin was measured using Rat/Mouse Insulin ELISA as per instructions (EMD Millipore, MA). The means were compared using one‐way ANOVA. Values are shown as standard error means ± (SEM)

### Intraperitoneal glucose and insulin tolerance tests (GTT/ITT)

Mice were anesthetized by brief inhalation of 2% isoflurane in pure oxygen, followed by an intraperitoneal injection of glucose (2.0 g/kg) or insulin (Humulin R; 0.75 U/kg), and blood was drawn from the tail at 0 (time point taken before injection), 15, 30, 60, and 120 min after injection with the OneTouch Ultra glucose monitor (LifeScan Inc.).

### Cholesterol measurements

At the end of the study, mice were euthanized and blood was collected via retro‐orbital sinus puncture. Cholesterol and triglycerides were measured from whole blood samples with a CardioCheck Plus Analyzer (PTS Diagnostics, IN). Epididymal white adipose tissue (EWAT) was removed after each mouse was sacrificed by cutting the tissue distal to the epididymal blood vessel. The adipose tissues were then weighed.

### Wire‐myography

Mice were killed by an overdose of CO_2_ inhalation and mesentery were removed and placed in cold Kreb's ringer buffer (KRB) with the following composition (in mmol/L): 118.5 NaCl, 4.7 KCl, 2.5 CaCl_2_, 1.2 MgSO_4_, 1.2 KH_2_PO_4_, 25.0 NaHCO_3_, and 5.5 D‐glucose. From the mesentery, three segments (2 mm) of second order mesenteric artery (MA_2_) from each mouse were carefully dissected. Segments were mounted in a wire myograph (model 620M; Danish Myotechnology, Aarhus, Denmark) for the recording of isometric force development. Segments were passively stretched according to a procedure first described by Halpern and Mulvany (Halpern and Mulvany 1977). In brief, segments were distended stepwise, in 50 μm increments to their optimal lumen diameter for active tension development. Segments were stretched to a passive wall tension of 90% of the internal circumference of that was achieved when they were exposed to a passive tension yielding a transmural pressure of 100 mmHg, which is referred to as its “optimal diameter.” At this passive wall tension, arteries were contracted with high K^+^ KRB (60 mmol/L KCl in KRB solution; replacing equimolar NaCl with KCl), thus generating a stable contraction that reached a plateau after 10–15 min. This active wall tension was set to a 100% contraction level. Contractile responses in MA_2_ were assessed by analyzing cumulative concentration‐response curves (CRCs) with the α_1_‐adrenergic agonist Phenylepinephrine (PHE; 0.01–30 μmol/L). Relaxing responses were determined in PHE (3–10 μmol/L) contracted segments by performing CRCs to Acetylcholine (ACh; 0.01–10 μmol/L). In a subset of MA_2_, NO‐dependent relaxations were analyzed in conditions where NO release and the endothelium‐dependent hyperpolarization (EDH) relaxing response was pharmacologically blocked by a cocktail of the cyclooxygenase blocker indomethacin (INDO; 10 μmol/L) and the endothelial K_Ca_ channel blockers TRAM‐34 and UCL1684 (both 1 μmol/L). EDH relaxing responses were assessed in the presence of the non‐selective NO synthase inhibitor L‐NAME and INDO. To study the role of endogenous peroxinitrite (OONO^−^) on EDH responses, MA_2_ were incubated with ebselen, a peroxinitrite scavenger (Masumoto and Sies 1996). To assess the effect of HFS diet on endothelium‐independent relaxations we first incubated MA_2_ with L‐NAME and INDO for 30 min to block endothelium‐dependent relaxations. Next, we used NO donor SNP (0.0001–1 μmol/L) in these arteries to determine ACh‐mediated relaxing responses in smooth muscle cells that might have been modulated due to HFS diet.

### Pressure myography

A segment (4 mm) of the MA_2_ was cannulated between two small glass micropipettes (O.D. 120–150 μm) in a pressure‐myograph chamber (model 110P, DMT‐USA, Inc., Aarhus, Denmark). The ends of the segment were tied with two small nylon knots (17 μm thin). The chamber was filled with warm Kreb's ringer buffer (KRB) and continuously gassed with carbogen. Intraluminal pressure was set to 60 mmHg and the artery was allowed to adjust in KRB containing 2.5 mmol/L CaCl_2_ for 30 min. The internal lumen diameter and wall thickness were continuously monitored using the MYOVIEW II software (DMT‐USA, Inc.). Viability was confirmed by adding the α_1_‐adrenergic agonist (PHE;0.1–1 μmol/L) to the myograph chamber. After a stable diameter was reached, cumulative concentrations of ACh (0.001–100 μmol/L) were added to the chamber. The chamber was washed three times with KRB and myogenic tone was assessed by monitoring internal diameter during stepwise (10 mmHg) increases in intraluminal pressures from 10 to 140 mmHg. At each pressure step, the artery was allowed to adjust its internal diameter for at least 2 min or until a stable diameter was obtained. Elastic characteristics of the vessel wall were determined under passive (calcium‐free) conditions in KRB without CaCl_2_ and containing 2.5 mmol/L ethylene glycol tetracetic acid (EGTA) and the NO donor SNP (10 μmol/L) to allow complete relaxation of the smooth muscle. According to Laplace's law, wall tension (*T* in mN/mm) depends upon transmural pressure (*P*_t_ in mN/mm^2^, where 100 mmHg equals 13.33 mN/mm^2^) and the radius of the artery (*r* in mm): *T *= *P*_t_ ∙ *r*. Circumferential wall stress (*σ* in mN/mm^2^) is related to wall thickness (*wt* in mm) and wall tension (*T*): *σ *= *T*/wt. Circumferential wall strain was calculated as (*D*_i_ – *D*_10_)/*D*_10_ where *D*_i_ is the lumen diameter at any intraluminal pressure and *D*_10_ the lumen diameter at 10 mmHg pressure. The incremental elastic modulus (*E*_inc_) was calculated according to Bergel (Bergel 1961). *E*_inc_ = 1.5 • *r*_o_^2^ • *r*_i_ • ΔP • (*r*_o_^2^ – *r*_i_^2^) • Δ*r*_i_^2^, where *r*_o_ = outer radius, *r*_i_ = inner radius, and ΔP = 10 mmHg or 1.333 mN/mm^2^. Cross‐sectional compliance (CC in μm^2^/kPa) was calculated as the change in inner lumen area (ΔA_i_) induced by a pressure change (ΔP_i_).

### Statistical analysis

Data were analyzed using one‐way analysis of variance (ANOVA) for comparison of multiple means followed by Tukey's post hoc test. Two‐way ANOVA, was used for concentration‐response curves followed by Bonferroni post hoc test (Prism‐Graphpad v6.0). Student's *t*‐test was performed for comparison of two means using Prism v 6.00 (GraphPad Prism software, La Jolla, CA). Data were represented as standard error of mean (SEM). The minimum number of animals used for experiments performed during this study was 6.

## Results

### Effect of HFS diet on weight gain, glucose tolerance, cholesterol, and triglyceride levels

**Figure 2:**
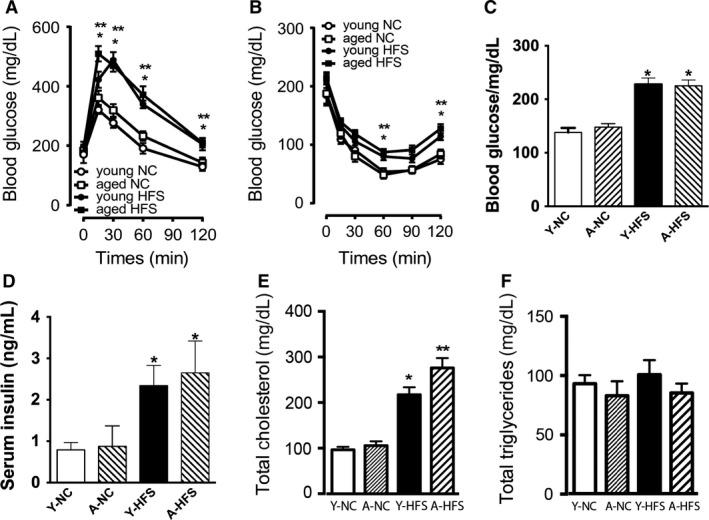
Effect of HFS on glucose intolerance, insulin resistance and cholesterol levels in young and aged mice: Glucose tolerance test at 4 months (young) and at 15 months (aged) (A), Insulin tolerance test at same age mentioned for ‘A’ (B), fasting blood glucose level of young and aged mice, (C), serum insulin levels (D), total cholesterol in whole blood (E), total triglycerides in whole blood (F), derived from mice receiving 6 months of normal chow (NC young; white bars), 16 months of NC (NC aged; dark gray bars), 3 months of HFD followed by 4 weeks of 10% sucrose in drinking water (HFS young; black bars), and 12 months of HFS (HFS aged; black bars). Values are shown as means ± SEM (*n* = 6); *P <* *0.05 versus young NC; **P <* *0.05 versus aged NC. *n* = 6; *P < 0.05 Young NC versus young HFS, ** Aged NC versus aged HFS.

The duration of feeding to four mice groups are presented in Figure 1A. Young mice on a NC diet for 4 months had significantly lower bodyweight (BW) than mice fed a HFS diet (Fig. 1B). There was no significant difference in the initial BW of both mice groups, but BW gradually increased and reached statistical significance (*P* < 0.01) after 6 weeks on a HFS diet (Fig. 1B). The BW measured after the end of the experiment was significantly higher (*P* < 0.01) in young or aged mice on HFS compared to young or aged mice on NC (Fig. 1C). Mice fed with HFS diet showed significant increase in EWAT (Fig. 1D) and also increased fat accumulation in the visceral cavity (not shown). Since obesity is directly linked to glucose homeostasis, we determined glucose tolerance levels between young HFS mice and age‐matched control NC fed mice, and also between aged NC mice and aged HFS mice with a glucose tolerance test (GTT). As shown in Figure 2A, HFS fed mice had impaired glucose tolerance compared with their age‐matched controls on NC. Additionally, aged HFS mice showed a higher glucose retention level compared to aged NC mice (Fig. 2A). However, there was no significant difference in glucose levels between young NC or aged NC mice, indicating no effect of age on glucose tolerance. We further determined insulin sensitivity in HFS or NC mice with an insulin tolerance test (ITT). When young or aged mice were given 0.75 U/kg of insulin after a 6‐h fasting period, the blood glucose of mice that were on a HFS diet (both young and aged) showed higher blood glucose retention at 60 and 120 min after the insulin injection compared with mice that were fed a NC diet (Fig. 2B), suggesting that young or aged HFS mice have developed significant insulin resistance. Supporting this finding we found that young or aged HFS mice had increased fasting glucose (Fig. 2C) and serum insulin levels (Fig. 2D) compared with age‐matched control young or aged NC mice. We also determined whether HFS diet would impact the cholesterol and triglyceride levels, as their increase is implicated in cardiovascular diseases. As shown in Figure 2E, young or aged HFS mice had an increase in cholesterol level compared with young or aged mice on NC respectively. However, there was no significant difference in the total triglycerides levels (Fig. 2F).

### Effect of HFS diet on small mesenteric artery remodeling and vasodilation

**Figure 3:**
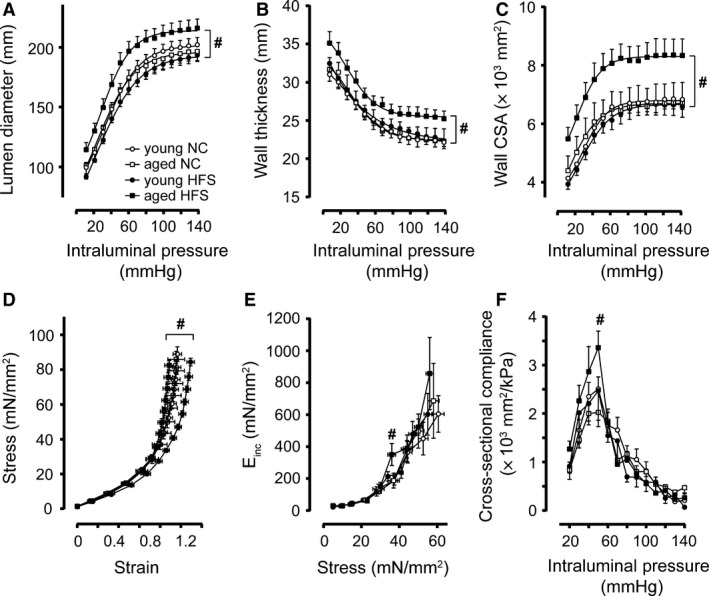
Age and HFS –mediated structural wall remodeling of small mesenteric arteries: Pressure–lumen diameter relationships (A), pressure ‐ wall thickness relationships (B), pressure–wall cross‐sectional area (CSA) relationships (C), circumferential wall strain – circumferential wall stress relationships (D), incremental elastic modulus – circumferential wall stress relationships (E), pressure – cross‐sectional compliance (F) for small mesenteric arteries derived from mice receiving 4 months of normal chow (NC young; open circles), 12 months of NC (NC aged; open squares), HFS young, closed circles, and 12 months of HFS (HFS aged; closed squares). Values are shown as means ± SEM (*n* = 6); *P <* *0.05 versus young NC; #*P *<* *0.05 versus young HFS.

**Figure 4:**
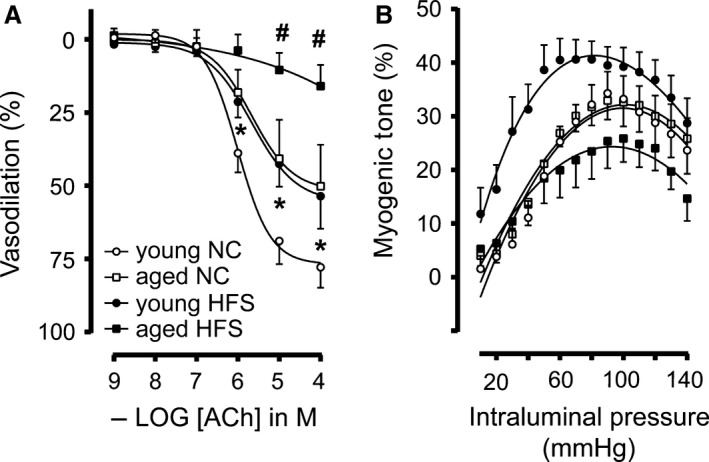
Effect of age and HFS on vasodilatation and myogenic tone in small mesenteric arteries: Endothelium‐dependent ACh‐mediated vasodilatation (A), and myogenic constriction (B) for small mesenteric arteries derived from mice receiving 4 months of normal chow (NC young; open circles), 12 months of NC (NC aged; open squares), HFS young (closed circles), and HFS aged (closed squares). Values are shown as means ± SEM (*n* = 6); *P <* *0.05 versus young NC; #*P *<* *0.05 versus young HFS.

We used pressure myography to determine the effect of HFS on resistance artery wall stress and wall thickness since lumen diameter and arterial wall remodeling would impact vascular flow dynamics. We determined the lumen diameter and wall thickness of MA_2_ that were cannulated and pressurized in the pressure‐myograph chamber. As shown in Figure 3A, the lumen diameters were significantly larger in MA_2_ from aged mice on a HFS diet compared to MA_2_ from young mice on HFS. Aging alone did not alter lumen diameter in mice on a normal chow diet (Fig. 3A). Similarly, wall thickness was larger in MA_2_ from aged HFS mice compared to their younger counterparts (Fig. 3B). Consequently, wall cross‐sectional area (CSA) of the MA_2_ was significantly increased in aged HFS group compared with younger HFS group (Fig. 3C), which indicated outward hypertrophic remodeling in response to a 1‐year HFS diet. Aging alone did not cause any significant changes in wall CSA in MA_2_ from NC mice (Fig. 3C). We calculated circumferential wall strain and circumferential wall stress values and plotted them against each other. Figure 3D showed that the slope of the strain–stress curves increased in MA_2_ from aged mice on a HFS diet compared to young mice on a HFS diet, indicating decreased arterial elasticity in the former. There was no significant difference between the stress strain curves from young or aged mice on a NC diet (Fig. 3D). At higher circumferential wall stress levels incremental elastic modulus (*E*_inc_) values were higher for MA_2_ from aged mice on a HFS diet compared to the other groups, again indicating an increased arterial stiffness during a near‐lifelong HFS diet (Fig. 3E). Due to the observed outward remodeling in MA from aged mice on a HFS diet, the cross‐sectional compliance was higher at low pressures, but dropped faster after 50 mmHg due to increased stiffness compared to the other groups (Fig. 3F). We also determined endothelium‐dependent ACh‐induced vasodilation in these arteries using wire myography. As shown in Figure 4A, ACh‐induced vasodilation was significantly impaired in MA_2_ from young HFS compared with young NC mice. However, impairment in ACh‐induced vasodilation in aged NC mice was similar to young HFS mice (Fig. 4A). In addition, aged HFS mice showed significantly decreased vasodilation compared to aged NC mice (Fig. 4A). These studies indicate that increased arterial stiffness and decreased relaxing responses in these arteries could have caused impaired vasodilation. Next, we determined the myogenic tone (pressure‐induced constriction) in these arteries using pressure myography to evaluate the effect of HFS on myogenic tone which might contribute to impaired vasorelaxation in these arteries. As shown in Figure 4B, myogenic tone was decreased in MA_2_ from aged HFS compared with young HFS, but did not reach statistical significance. This indicates a tendency toward impairment of constrictor tone (Fig. 4B). Taken together, decreased elasticity and increased stiffness of MA_2_ was observed in mice with HFS, indicating that structural elements of resistance arteries might undergo HFS‐mediated remodeling.

### Effect of HFS on small mesenteric artery endothelial dysfunction in young and aged mice

**Figure 5:**
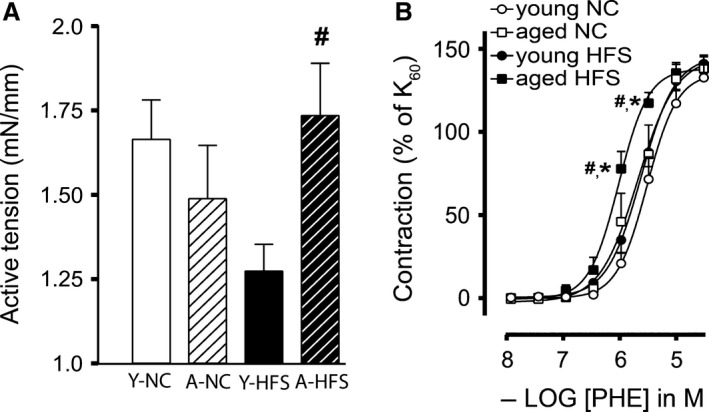
Effect of age and HFS on contractile response of small mesenteric arteries: Active tension (mN/mm) in response to 60 mmol/L KCl in KRB (A), cumulative concentration‐response curves (CRC) to the α_1_‐adrenergic receptor agonist phenylephrine (B) for small mesenteric arteries derived from mice receiving 4 months of normal chow (NC young; open circles), 12 months of NC (NC aged; open squares), HFS young (closed circles), and HFS aged (closed squares). Values are shown as means ± SEM (*n* = 6–9); *P <* *0.05 versus young NC; #*P *<* *0.05 versus young HFS.

**Figure 6:**
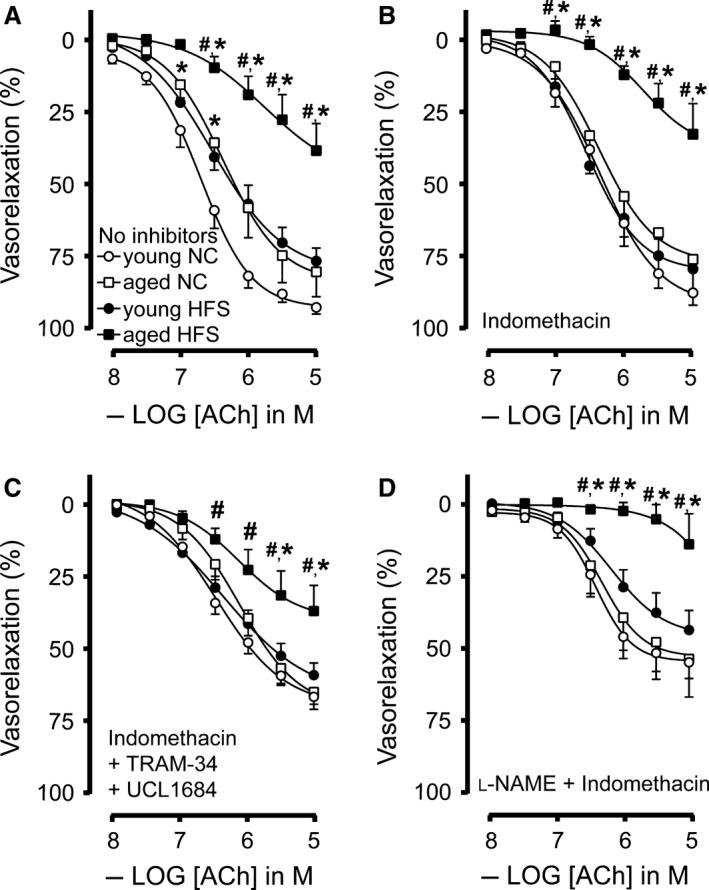
Effect of age and HFS on endothelium‐dependent relaxation of small mesenteric arteries: Endothelium‐dependent ACh‐mediated relaxations in the absence of any inhibitors (A), endothelium‐dependent ACh‐mediated relaxations in the presence of the non‐selective cyclooxygenase blocker indomethacin (B), NO‐dependent ACh‐mediated relaxations in the presence of indomethacin, TRAM‐34 and UCL 1684 (C), endothelium‐dependent hyperpolarizing (EDH) relaxing responses in the presence of indomethacin and the non‐selective NOS blocker L‐NAME (D) for small mesenteric arteries derived from mice receiving 3 months of normal chow (NC young; open circles), NC aged (open squares), HFS young (closed circles), and HFS aged (closed squares). Values are shown as means ± SEM (*n* = 6–9); * *P *<* *0.05 versus young NC; # *P *<* *0.05 versus young HFS.

Since increased arterial stiffening and decreased arterial elasticity of MA_2_ was observed in response to HFS diet, we determined the impact of these arterial structural changes on endothelial dysfunction in HFS diet. Vascular contractile and relaxing responses were measured using wire myography. Vascular contractile responses to 60 mmol/L KCl were decreased in MA_2_ from young HFS mice compared with young NC (Fig. 5A). However, contractile tension in response to this depolarizing solution was significantly increased in MA_2_ from aged HFS compared with young HFS (Fig. 5A). Contractile responses to the α_1_‐adrenergic agonist PHE were significantly increased in MA from aged mice on a HFS diet compared to the other groups (Fig. 5B). Similar to observations in the pressure‐myograph, relaxations to ACh were significantly blunted in MA_2_ from young HFS compared with age‐matched NC, whereas a combination of aging and HFS diet had a more severe inhibitory effect on endothelium‐dependent ACh‐mediated relaxations (Fig. 6A). Sensitivity (pEC_50_) and maximal relaxing responses (*E*_max_) to ACh are summarized in Table 1. Interestingly, endothelium‐dependent relaxing responses from MA_2_ of aged NC mice did not differ from young HFS mice (Fig. 6A), suggesting that a HFS diet can accelerate endothelial dysfunction in young mice similar to aged mice in NC. The endothelial dysfunction caused by a four‐month HFS diet was due to increased release of vaso‐contractile prostaglandins because the nonselective cyclooxygenase blocker indomethacin restored ACh‐induced relaxing responses in MA_2_ from young HFS (Fig. 6B and Table 1). Surprisingly, prolonged HFS diet for 12 months caused an irreversible endothelial dysfunction that could not be restored by indomethacin (Fig. 6B and Table 1). Microvessels such as MA_2_ are known to show EDHF‐mediated vasorelaxation, and to a lesser degree, the NO‐dependent vasodilation (Takaki et al. 2008). We sought to determine the relative contribution of NO or EDHF on endothelial dysfunction in HFS fed mice. We first used a cocktail of Indomethacin, TRAM34 and UCL1684 to inhibit prostaglandins and endothelial calcium activated K^+^ channels, respectively to block EDHF‐mediated relaxation. As shown in Figure 6D, endothelium‐dependent (NO‐dependent) ACh‐mediated relaxations were impaired in aged HFS mice, but not in young HFS mice. Next, we blocked NO‐mediated relaxing responses using eNOS blocker, L‐NAME and non‐selective cyclooxygenase inhibitor indomethacin, and determined ACh‐mediated relaxations to determine the contribution of EDHF in small vessel relaxation in HFS. As shown in Figure 6D, an acute impairment of EDHF‐dependent relaxation was observed in aged HFS fed mice, but not in aged NC or young NC mice. Young HFS mice showed a mild decrease in relaxation compared to young NC mice, but this was not statistically significant. These data show that although age and HFS‐induced endothelial dysfunction was characteristic of both, an impaired NO release (Fig. 6C) and a reduced endothelium‐dependent hyperpolarization (Fig. 6D), EDHF response was a major relaxing factor in HFS fed aged mice.

**Table 1:** Effect of pharmacological inhibitors on sensitivity (pEC_50_) and maximal relaxing responses (*E*_max_) to ACh in mesenteric resistance arteries from young and aged mice on a normal chow (NC) and a high fat sucrose (HFS) diet

Treatment	Parameter	NC young	NC aged	HFS young	HFS aged
No inhibitors	pEC_50_ (−log M)	6.60 ± 0.05	6.05 ± 0.09*	6.04 ± 0.07*	4.66 ± 0.14* ^,^ #
	*E*_max_ (%)	91 ± 2	79 ± 8	75 ± 4*	37 ± 9* ^,^ #
I	pEC_50_ (‐ log M)	6.19 ± 0.06**	5.90 ± 0.13	6.13 ± 0.08	4.59 ± 0.21* ^,^ #
	*E*_max_ (%)	86 ± 4	74 ± 12	78 ± 5	32 ± 10* ^,^ #
T+U+I	pEC_50_ (−log M)	5.79 ± 0.05**	5.60 ± 0.07**	5.54 ± 0.07**	4.67 ± 0.23* ^,^ #
	*E*_max_ (%)	67 ± 3**	66 ± 6**	60 ± 4**	37 ± 9* ^,^ #
L‐N+I	pEC_50_ (−log M)	5.49 ± 0.11**	5.36 ± 0.18**	4.96 ± 0.09**	4.27 ± 0.90* ^,^ #
	*E*_max_ (%)	55 ± 6**	54 ± 14**	42 ± 7**	13 ± 11* ^,^ ** ^,^ #
L‐N+I+ebselen	pEC_50_ (−log M)	5.74 ± 0.13**	5.97 ± 0.19	6.13 ± 0.06	5.61 ± 0.15**
	*E*_max_ (%)	72 ± 7**	67 ± 16	73 ± 4	63 ± 9*

The sensitivity (pEC_50_) of ACh is calculated with GraphPad Prism6 software as described in the methods and denotes the negative logarithmic concentration of ACh that induces 50% relaxation compared to the maximal relaxation (*E*_max_). Abbreviations used: I (indomethacin), L‐N (L‐NAME), T (TRAM‐34), and U (UCL1684). See text for inhibitor concentrations used. Values are shown as means ± SEM. *P <* *0.05 versus NC Young, #*P *<* *0.05 versus HFS Young, **P <* *0.05 versus no inhibitors.

### Effect HFS diet on EDHF‐mediated endothelial dysfunction in young and aged mice

**Figure 7:**
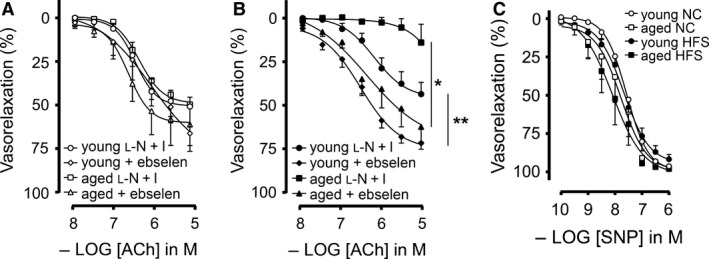
Effect of ebselen on EDH relaxing responses in small mesenteric arteries from young and aged mice in HFS: (A) Endothelium‐dependent hyperpolarizing (EDH) relaxing responses in the absence (L‐N+I) or presence of ebselen for small mesenteric arteries derived from young and aged mice receiving normal chow. (B) Endothelium‐dependent hyperpolarizing (EDH) relaxing responses in the absence (L‐N+I) or presence of ebselen for small mesenteric arteries derived from young and aged mice receiving a high‐fat and high‐sucrose diet. Values are shown as means ± SEM (*n* = 6); *P <* *0.05 aged L‐N+I versus aged + ebselen; **P <* *0.05 young L‐N+I versus young + ebselen; (C) Endothelium‐independent sodium nitroprusside (SNP; 0.1 nmol/L–1 μmol/L)‐mediated relaxations in the presence of L‐NAME and indomethacin for small mesenteric arteries derived from mice receiving 3 months of normal chow (NC young; open circles), NC aged (open squares), HFS young (closed circles), and HFS aged (closed squares). Values are shown as means ± SEM (*n* = 6–9).

In a recent publication we have shown increased production of O_2_^•−^ in the thoracic artery of aged mice (Hilgers et al. 2017). Dysfunctional eNOS was the major contributor for enhanced O_2_^•−^ generation. Others and we have also shown increased ROS generation in response to hyperglycemia and mice fed with HFD (Patel et al. 2013; Heinonen et al. 2014). We reasoned that O_2_^•−^ and NO produced in the aged HFS fed mice would react to produce OONO^‐^ that might decrease the levels of H_2_O_2_ causing an impairment of EDHF mediated vasorelaxation, as H_2_O_2_ has been shown as an EDHF in mesenteric vascular bed (Matoba et al. 2000). Therefore, if OONO^‐^ was a major inhibitor of EDH response, then we could rescue this response using ebselen, a potent peroxinitrite scavenger (Masumoto and Sies 1996). As shown in Figure 7A, ebselen (0.2 μmol/L) did not have a significant effect in improving EDH responses in MA_2_ from young or aged mice on NC diet. However, ebselen markedly ameliorated EDH responses in MA_2_ from both young and aged mice on a HFS diet (Fig. 7B). In fact, EDH responses were normalized for all 4 mice groups (Table 1), suggesting that ONOO^‐^ production might have contributed to endothelial dysfunction in HFS fed mice. We also determined the endothelium‐independent relaxations to NO donor SNP to demonstrate the effect of HFS‐mediated endothelial dysfunction specifically in the endothelium, but not in the smooth muscle cells. As shown in Figure 7C, relaxing responses to the NO donor SNP were unchanged in either group, demonstrating normal smooth muscle NO sensitivity as SNP produces NO via soluble gluanylyl cyclase (sGC) in the smooth muscle cells.

## Discussion

In this study we have utilized an obesity model (young NC vs. young HFS), an aging model (young NC vs. aged NC), and an aging plus obesity model (young HFS vs. aged HFS). The goal was to determine the effect of obesity on young, as well as aged using the same group of mice as they age. We have shown that metabolic disorder (such as T2D) induced by HFS‐mediated obesity adversely affects vascular functions, which is accentuated as the animal ages. We have shown that obesity is associated with: (1) impaired glucose and insulin tolerance with high blood glucose, high cholesterol levels. (2) metabolic impairment resulting in vascular endothelial dysfunction in resistance arteries, and (3) decreased myogenic tone in resistance arteries. We also showed that aging is associated with impaired ACh‐mediated vasodilation. However, there were no significant changes in weight gain, glucose or insulin levels, and glucose tolerance in young and aged groups. Furthermore, there was no alteration in myogenic tone or EDHF response between these groups. However, the aged obese mice showed significant: (1) increase in lumen diameter, arterial wall thickness and increased CSA; (2) decrease in arterial elasticity and increased arterial stiffness; (3) blunted vasodilation, decreased myogenic tone; (4) increase in contractile responses; (5) irreversible endothelial dysfunction that could not be restored by indomethacin; (6) impairment of EDHF‐dependent vasorelaxation that could be restored by ebselen.

Our study addressed both functional (contractile and relaxing responses) and structural (arterial remodeling and elasticity) characteristics of small mesenteric arteries derived from young or aged mice on a normal or high‐fat diet. Consumption of HFS diet resulted in significant weight gain, hypercholesterolemia, glucose intolerance, and insulin resistance. Indeed, C57BL/6J mice have been shown to be very sensitive to the development of obesity and T2D when placed on high‐fat diet (Rebuffe‐Scrive et al. 1993; Surwit et al. 1995; Collins et al. 2004). HFS diet in C57BL/6J mice resembles diet‐induced obesity and T2D as observed in humans. T2D has also an increasing correlation with age that was recapitulated in our aged mice with HFS diet. We studied C57BL/6J mice that received a normal diet and assessed microvascular reactivity and structure in young (7 months) and aged (15 months) mice. The purpose of these two groups was to dissociate the aging effect from the diet‐induced obesity effects.

### Short‐term effects of HFS diet on microvascular structure and function

Pressure myography was applied to study pressure‐induced myogenic constriction and arterial structural parameters, such as inner diameter, wall thickness, media CSA, strain–stress relationships, incremental elastic modulus, and cross‐sectional compliance. None of the abovementioned parameters were significantly altered in small mesenteric arteries from mice after a four‐month HFS diet compared with mice on a regular diet. We observed impaired endothelium‐dependent ACh‐induced relaxing responses in small mesenteric arteries that were fed a HFS diet for four months. This endothelial dysfunction is in agreement with other experimental obesity rodent models studying this vascular bed (O'Brien et al. 1998; Naderali et al. 2001; Subramanian and MacLeod 2003; Oltman et al. 2006; Young et al. 2008). Our results are in contrast to the study by Ellis and colleagues, who did not observe endothelial dysfunction in small mesenteric arteries in mice on a western diet for 20 weeks (Ellis et al. 2008). We assessed NO‐mediated relaxing responses in the presence of the cyclooxygenase blocker indomethacin (to rule out vasoactive prostaglandins) and the endothelial calcium‐activated K^+^ channel blockers TRAM 34 and UCL 1684 to inhibit EDH and subsequent smooth muscle relaxation. We did not observe a statistically significant reduction in NO‐mediated relaxing responses after four months of HFS diet. Besides NO, other important mechanisms contribute to relaxation in small resistance arteries, such as the EDH response. In the mesenteric vascular bed, the Ca^2+^‐activated small (K_Ca_2.3)‐ and intermediate (K_Ca_3.1)‐conductance K^+^ channels play a crucial role in the EDH response (Burnham et al. 2002; Hilgers and Webb 2007). Studies have shown that this EDH response mediated by K_Ca_3.1 and K_Ca_2.3 channels remains unaltered or even increased in obesity (Chadha et al. 2010; Climent et al. 2014). When small mesenteric arteries were treated with the NO synthase blocker L‐NAME and indomethacin, the residual EDH response tended to be lower after four months of HFS diet, but this did not reach statistical significance compared with mice given a normal diet. Endothelium‐dependent ACh‐induced relaxation was restored in the presence of indomethacin alone, suggesting an enhanced contribution of vasocontractile prostaglandins released in response to a HFS diet. Indeed, diminished release of arachidonic acid‐induced prostacyclin (PGI_2_) and increased thromboxane (TXA2)‐mediated contraction has been observed in obesity animal models (Hodnett et al. 2009; Gamez‐Mendez et al. 2015). Endothelium‐independent relaxations to the NO donor SNP were similar for all groups suggesting no alteration in smooth muscle function. Thus, HFS impairs vasorelaxation in an endothelial‐specific manner.

### Effects of aging on microvascular structure and reactivity

We observed endothelial dysfunction in small mesenteric arteries from aged mice on a normal diet compared with young mice on a normal diet, as characterized by a reduced sensitivity to ACh, but this impaired NO or EDH‐mediated relaxing responses was not statistically significant. Indomethacin potentiated the ACh‐induced response in small mesenteric arteries from aged mice on a NC diet, suggesting generation of vasocontractile prostanoids with aging, as shown by others in rat mesenteric arteries (Matz et al. 2000; Stewart et al. 2000).

It is known that ROS and cytokines, factors that are increased with aging, can induce the expression of inducible prostaglandin H synthase, which leads to vasoconstriction with aging rats (Stewart et al. 2000). However, we did not observe hyper reactivity to phenylephrine in aged mice, suggesting that the functional differences between young and aged vessels were the result of altered endothelial metabolism rather than changes in smooth muscle responsiveness, which was confirmed by the similar sensitivity to the NO donor SNP and comparable structural characteristics between small mesenteric arteries from young and aged mice on a normal diet. Endothelial dysfunction has been described with aging in both animals and humans in response to agonists or hyperemia. A reduced ACh‐mediated response was observed in both small and large arteries with aging (Hongo et al. 1988; Atkinson et al. 1994; Kung and Luscher 1995; Taddei et al. 1995). In our study using resistance arteries, which are also considered as microvessels, endothelium‐dependent ACh‐induced relaxing responses were similar between aged mice on a regular diet and young mice on the HFS diet, suggesting that HFS induces endothelial dysfunction in microvessels in young mice. Consistent with this observation using large conduit arteries such as aortae, Bailey‐Downs et al. (2013) have reported endothelial dysfunction in young mice on HFD, which was similar to aged mice on a standard diet.

### Long‐term deleterious effects of HFS diet

In response to long‐term HFS diet, small mesenteric arteries showed an outward hypertrophic remodeling characteristic of both an increased wall thickness and wall cross‐sectional area when compared with age‐matched mice fed a regular diet. In addition, a decrease in arterial elasticity was observed as evidenced by significant leftward shift of the stress–strain curve and increased incremental elastic modulus in aged mice fed on HFS diet compared with aged mice on a normal diet. Furthermore, small mesenteric arteries from aged mice on a HFS diet showed a tendency for decreased myogenic tone compared to small mesenteric arteries from age‐matched NC mice. Previous studies have reported that loss of myogenic tone is associated with altered EDHF response (McSherry et al. 2006), and therefore EDHF could be a major contributor of vasorelaxation in aged HFS mice. Similar myogenic response has also been observed by Ogalla, et al. in their studies with resistance arteries in ApoE^‐/‐^ mice fed with HFD (Ogalla et al. 2015)

Active tension in response to a depolarizing potassium chloride solution was increased in small mesenteric arteries from aged mice on HFD compared to their counterparts from aged‐matched mice on a NC. It is likely that the increase in lumen diameter and wall mass in the former generated a greater contractile force in response to the depolarization. We observed a severely blunted endothelium‐dependent ACh‐induced relaxing response in small mesenteric arteries from mice fed a HFS diet for 12 months. This was observed in both, pressurized arteries and stretched arteries. This endothelial dysfunction was characteristic of both an impaired NO‐ and EDH‐mediated response. The reduced NO bioavailability is characteristic of obesity (Naderali et al. 2001; Erdos et al. 2002; Frisbee et al. 2002). Increased production of vascular O_2_^•−^ has been shown to lead to reduced NO bioavailability, resulting in reduced agonist‐induced relaxing responses of mesenteric and skeletal muscle microvessels of obese mice and rats (Bohlen and Lash 1993; Frisbee and Stepp 2001; Bagi et al. 2003). This increase in O_2_^•−^ production might be responsible for the observed increase in sensitivity to phenylephrine in small mesenteric arteries from aged mice on a HFS.

Our study also showed a severely impaired EDH response in small mesenteric arteries from aged mice on a HFS diet compared with age‐matched mice on a regular diet. Other studies have observed unaltered or even a compensatory increase in the EDH response during obesity (Burnham et al. 2002; Ellis et al. 2008). These conflicting results may be vascular‐bed specific and could be explained by the fact that our experimental obesity model is accompanied by hyperglycemia, hypercholesterolemia, and insulin resistance; all of which may have additive deleterious effects on the endothelium. In addition, our temporal study shows the progressive nature of the severity of endothelial dysfunction, hypercontractility, and degree of outward hypertrophic remodeling in small mesenteric arteries. The peroxynitrite scavenger, ebselen, restored the EDH‐relaxing response, confirming enhanced ROS production during obesity by other studies (Erdei et al. 2006; Oltman et al. 2006). We propose to conduct future experiments to precisely understand the mechanism by which endothelial dysfunction occurs in a metabolic disease. We have already shown that vascular redox state is critical to endothelial function, and a shift of vascular redox state from reducing state to oxidizing state occurs in aging (Hilgers et al. 2017). This alteration causes age‐related hypertension. We hypothesize that metabolic disease such as T2D causes altered vascular redox conditions, which may produce a dysfunctional eNOS and also an impairment of EDHF (Hilgers and Das 2015)

One of the limitations in this study was a lack of ROS detection in the arteries of NC or HFS mice. However, we have recently shown that aged MA_2_ generates significant amounts of superoxide anion via a dysfunctional eNOS using electron paramagnetic resonance spectrometry (Hilgers et al. 2017). In addition to eNOS, activated NADPH oxidases (Nox) or mitochondria could also generate HFS‐mediated O_2_^•−^ (Garcia‐Ruiz et al. 2016; Zeng et al. 2017). The other limitation is the specificity of ebselen for scavenging OONO^‐^ as ebselen can scavenge other radicals.

## Conclusion

In summary, our study presents evidence that prolonged feeding of HFS diet promoted a T2D phenotype in young, as well as aged mice with glucose intolerance and higher blood glucose levels. These changes are associated with blunted endothelial dysfunction in young HFS mice. Additionally, HFS diet with aging exacerbated endothelial dysfunction. Furthermore, we showed that decreased EDHF in aged HFS fed mice is a major contributor to endothelial dysfunction as antioxidant ebselen restored the endothelial function in these mice. We conclude that prolonged HFS diet in aged mice causes endothelial dysfunction in small resistance arteries due to loss of EDHF, complicating glucose tolerance and glucose utilization that accelerates a T2D phenotype.
