# Decreased EDHF‐mediated relaxation is a major mechanism in endothelial dysfunction in resistance arteries in aged mice on prolonged high‐fat sucrose diet

**DOI:** 10.14814/phy2.13618

**Published:** 2018-02-11

**Shannon M. Dunn, Robert Hilgers & Kumuda C. Das**

Physiol Rep, 5 (23), 2017, e13502, https://doi.org/10.14814/phy2.13502

In Dunn et al. (2017), the author, Robert Hilgers, was left out from the author listing in the article, his name has been included after a post publication correction.

The authors apologise for the error.
